# Influenza A Virus Infections in Land Birds, People’s Republic of China

**DOI:** 10.3201/eid1410.080169

**Published:** 2008-10

**Authors:** A. Townsend Peterson, Sarah E. Bush, Erica Spackman, David E. Swayne, Hon S. Ip

**Affiliations:** University of Kansas, Lawrence, Kansas, USA (A.T. Peterson, S.E. Bush); US Department of Agriculture, Athens, Georgia, USA (E. Spackman, D.E. Swayne); US Geological Survey, Madison, Wisconsin, USA (H.S. Ip)

**Keywords:** Avian influenza, land birds, prevalence, China, dispatch

## Abstract

Water birds are considered the reservoir for avian influenza viruses. We examined this assumption by sampling and real-time reverse transcription–PCR testing of 939 Asian land birds of 153 species. Influenza A infection was found, particularly among migratory species. Surveillance programs for monitoring spread of these viruses need to be redesigned.

Avian influenza virus ecology has long regarded waterbirds as a primary reservoir. Although the benchmark study detailed prevalences across all taxa (*1*), subsequent studies have focused exclusively on waterbirds (*2*) with few exceptions (*3*,*4*). We reexamined these assumptions on the basis of a broad sampling of bird diversity in Southeast Asia, where bird-borne influenza viruses are of particular concern (*5*). We sampled and tested diverse land birds for influenza A virus infection and showed that land birds also harbor infections with these viruses. Birds in these taxa are not irrelevant in virus transmission and should form an integral part of avian influenza surveillance and monitoring programs.

## The Study

During 2004–2007, as part of a broader biodiversity survey and inventory program, we sampled birds from mostly forested sites in Guangxi and Guizhou Provinces in the southern part of the People’s Republic of China (Figure). Sampling was conducted by mist netting and selective harvesting with shotguns; all birds in the study were apparently healthy and behaving normally at the time of collection. Because initial sampling was focused on endoparasite communities, samples from 2004–2005 consisted of complete gastrointestinal tracts frozen in liquid nitrogen. In 2006–2007, sampling was conducted specifically for viruses. Cloacal swabs were collected in 2006 and buccal–cloacal swabs were collected in 2007. All swabs were preserved in 95% ethanol.

**Figure:**
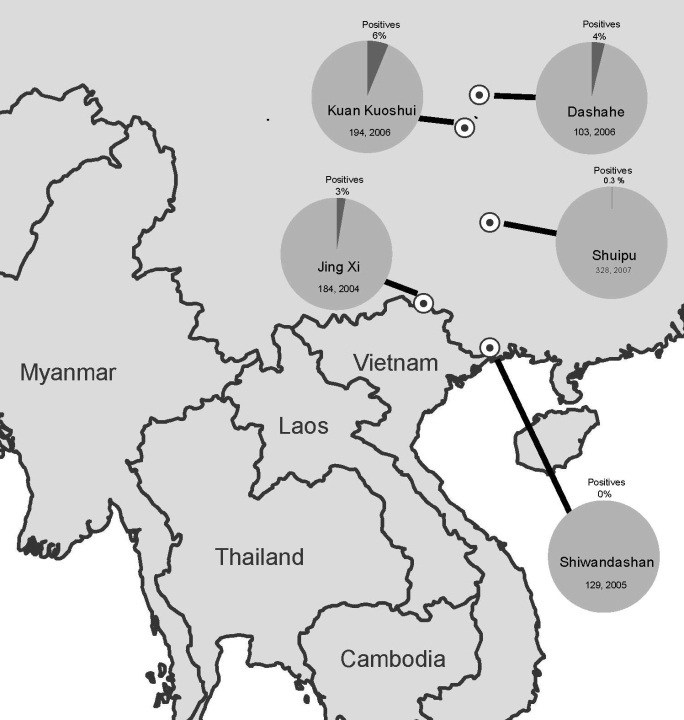
Southeastern Asia, showing 5 sites in the People’s Republic of China where land birds were collected and tested for influenza A virus. Prevalence values were 4% (n = 103) in Dashahe in 2006; 6% (n = 194) in Kuan Kuoshui in 2006; 0.3% (n = 328) in Shuipu in 2007; 3% (n = 184) in Jing Xi, in 2004; and 0% (n = 130) in Shiwandashan in 2005.

A total of 184 samples were collected from Jing Xi municipality in Guangxi (21.122°N, 105.964°E) in 2004, 130 from Shiwandashan Nature Reserve in Guangxi (21.840°N, 107.880°E) in 2005, 103 from Dashahe Nature Reserve in Guizhou (29.167°N, 107.575°E) in 2006, 194 from Kuan Kuoshui Nature Reserve in Guizhou (28.226°N, 107.160°E) in 2006, and 328 from Shuipu village, Guizhou Province (25.485°N, 107.882°E) in 2007 (Figure). Samples were tested for influenza A virus by real-time reverse transcription–PCR (*6*) in 2 diagnostic laboratories (Southeast Poultry Research Laboratory, US Department of Agriculture, Athens, GA, USA, and National Wildlife Health Center, US Geological Survey, Madison, WI, USA).

Of 939 samples tested, 24 were positive for influenza A viruses (prevalence 2.3%, Table; complete summary in Appendix Table). If migratory behavior (species classified as migratory or nonmigratory on the basis of descriptions by MacKinnon and Phillipps [*7*]), was considered, 11 (4.8%) of 231 samples from species showing marked seasonal migrations were influenza positive. However, only 13 (1.8%) of 708 samples from nonmigratory species were positive. The cumulative binomial probability that such a high number (*11*) of positive samples would result among the 231 migratory-species samples, were the true prevalence to be 1.8%, is low (p = 0.0013). Thus, migratory species appear to have higher influenza infection rates. In terms of general habitat use (*7*), open-country species were slightly more prone to be influenza positive (8 [2.9%] of 274 samples) than forest species (16 [2.4%] of 665 samples), but the difference was not significant (cumulative binomial probability, p>0.05). Interactions between migratory behavior and habitat use were not significant (contingency test, p>0.05). Although all infections detected were among songbirds (Passeriformes), the sampling also concentrated on songbirds (94.3%). Thus, we could not test adequately the hypothesis that influenza prevalence was equivalent between songbirds and other birds.

**Table:** Prevalence of influenza A virus in avian orders and families at 5 sites, People’s Republic of China

Order	Family	Location, no. positive/no. tested				
		Dashahe	Jing Xi	Kuan Kuoshui	Shiwandashan	Shuipu
Apodiformes	Apodidae	0/4				
Caprimulgiformes	Caprimulgidae				0/2	0/1
Charadriiformes	Scolopacidae		0/1			
Ciconiiformes	Ardeidae		0/1		0/1	
Columbiformes	Columbidae	0/1	0/1	0/1	0/1	
Coraciiformes	Alcedinidae		0/1		0/1	0/4
Cuculiformes	Cuculidae			0/1	0/1	
Gruiformes	Rallidae					0/1
Passeriformes	Aegithalidae	0/3				0/11
	Aegithinidae		0/1			0/2
	Campephagidae	0/1	0/4	0/1	0/8	0/4
	Cinclidae	0/5				
	Corvidae	0/2	0/1	0/3		
	Dicaeidae				0/6	
	Dicruridae		0/8		0/1	
	Emberizidae	0/10		3/18		0/20
	Estrildidae					0/13
	Fringillidae			0/1		0/6
	Hirundinidae					0/3
	Laniidae					0/1
	Monarchidae		0/6		0/8	
	Motacillidae	0/3	1/4	0/1		0/12
	Muscicapidae	2/18	2/42	1/26	0/31	1/55
	Nectariniidae			0/1	0/7	0/2
	Panuridae	0/2	0/1	0/11		0/6
	Paridae	0/2	0/1	1/20		0/10
	Passeridae	1/1		1/1		0/11
	Pycnonotidae	0/9	0/8	0/4	0/18	0/47
	Sturnidae	0/1				
	Sylviidae	1/20	1/34	2/21	0/13	1/20
	Timaliidae	0/20	1/64	3/76	0/25	1/76
	Turdidae					0/2
	Zosteropidae			1/1	0/1	0/11
Piciformes	Capitonidae				0/1	
	Picidae	0/1	0/5	0/6	0/4	0/11
Podicipediformes	Podicipedidae			0/1		
Trogoniformes	Trogonidae		0/1			

An obvious question is whether the influenza A viruses we detected belong to the highly pathogenic subtype H5N1 strain currently circulating across much of Asia. All samples were negative for the H5 subtype by real-time reverse transcription–PCR (*6*), although this result does not exclude the possibility that H5 viruses were among the positive samples. The preservation status of samples we tested prevented virus isolation or full, strain-level characterization of influenza viruses.

## Conclusions

The subtype H5N1 strain of influenza virus has spread rapidly and has been detected across much of central and southern Eurasia. Although movements of wild birds have been implicated in this spread (*8*), other studies question (*9*,*10*) or contradict (*11*) this idea. An important part of the argument centers on the question of the occurrence of the virus in wild birds without obvious illness, which can be difficult to interpret given the low prevalence of influenza. For instance, a recent study based on sampling >13,000 migratory birds in China detected the subtype H5N1 strain of influenza virus only 8 times (*12*), and similar results have been obtained elsewhere (*2*). Our study, although not successful in characterizing influenza viruses to specific strains, nonetheless shows that influenza A virus infection occurs in more bird species than previously assumed and that influenza A infections can be found in birds that behave normally and show no sign of illness.

Although a review of avian influenza virus ecology (*1*) discussed the occurrence of influenza viruses across all groups of birds (and other vertebrates), subsequent studies have assumed that waterbirds are the primary reservoir (*8*,*13*,*14*). In this study, a broad sample of land birds yielded frequent influenza-positive results. Although waterbirds could have higher prevalences, we have demonstrated broad occurrence of influenza viruses in diverse taxa of Passeriformes (songbirds) in Southeast Asia. This result suggests that land birds may also be a major reservoir of influenza viruses.

We have taken a step toward a more complete understanding of influenza virus ecology among wild birds. Our partial survey of influenza virus distributions across the rich avifaunas of the southern region of China demonstrated frequent infections. This result contrasts with the current dogma in the influenza surveillance community. We suggest that to be effective future surveillance efforts will need to include the full diversity of wild birds.

## Supplementary Material

Appendix Table Summary of individual birds tested, People's Republic of China*
