# Supplementary material for: Influenza A Virus Infections in Land Birds, People’s Republic of China
Source: Emerg Infect Dis. 2008 Oct;14(10):1644–6. doi: 10.3201/eid1410.080169 (PMC2609895; doi:10.3201/eid1410.080169)
Supplement: Appendix Table — Summary of individual birds tested, People's Republic of China* [file 08-0169_appT-s1.pdf]

Appendix Table. Summary of individual birds tested, People's Republic of China*

Order	Family	Scientific name	Site	KUNHM	No. positive	Sample	Status	Location
Apodiformes	Apodidae	*Apus pacificus*	Dashahe	97415, 97621,98120, 98940	0	4	M	O
Caprimulgiformes	Caprimulgidae	*Caprimulgus indicus*	Shiwandashan	96345, 96346	0	2	M	O
			Shuipu	99676	0	1	M	O
Charadriiformes	Scolopacidae	*Gallinago rusticola*	Jing Xi	96395	0	1	M	O
Ciconiiformes	Ardeidae	*Butorides striatus*	Jing Xi	93320	0	1	M	O
		Gorsachius melanolophus	Shiwandashan	96393	0	1	M	F
Columbiformes	Columbidae	*Chalcophaps indica*	Jing Xi	93308	0	1	R	F
			Shiwandashan	96422	0	1	R	F
		Streptopelia chinensis	Dashahe	97413	0	1	R	O
		S. orientalis	Kuan Kuoshui	97407	0	1	R	O
Coraciiformes	Alcedinidae	*Alcedo hercules*	Jing Xi	93313	0	1	R	F
		A. atthis	Shuipu	99683, 99684, 99685, 99956	0	4	R	O
		Ceyx erithacus	Shiwandashan	96412	0	1	R	F
Cuculiformes	Cuculidae	*Cuculus micropterus*	Kuan Kuoshui	97406	0	1	R	F
		Phaeniocophaeus tristis	Shiwandashan	96418	0	1	R	F
Gruiformes	Rallidae	*Amaurornis phoenicurus*	Shuipu	99677	0	1	R	O
Passeriformes	Aegithalidae	*Aegithalos concinnus*	Dashahe	97478, 97620, 98936	0	3	R	F
			Shuipu	99423, 99424, 99760, 99761, 99762, 99763, 99764, 99765, 99948, 99963, 1 not catalogued	0	11	R	F
	Aegithinidae	*Chloropsis hardwickii*	Jing Xi	93411	0	1	R	F

		Shuipu	99686, 99687	0	2	R	F
Campephagidae	*Coracina macei*	Jing Xi	93314, 93315	0	2	R	F
	C. melaschistos	Jing Xi	96238	0	1	R	F
		Kuan Kuoshui	97461	0	1	R	F
	Hemipus picatus	Jing Xi	96587	0	1	R	F
		Shiwandashan	96512	0	1	R	F
	Pericrocotus divaricatus	Shiwandashan	96485, 96486, 96487	0	3	M	F
	P. ethologous	Dashahe	97433	0	1	R	F
	P. flammeus	Shiwandashan	96488, 96489, 96490, 96491	0	4	R	F
		Shuipu	99945, 99691	0	2	R	F
	P. solaris	Shuipu	99945, 99691	0	2	R	F
Cinclidae	*Cinclus pallasii*	Dashahe	97487, 97488, 98937, 99345, 99359	0	5	R	F
Corvidae	*Dendrocitta formosae*	Jing Xi	93319	0	1	R	F
	Garrulus glandarius	Kuan Kuoshui	97526	0	1	R	F
	Urocissa erythrorhyncha	Dashahe	1 not catalogued, 97424	0	2	R	O
		Kuan Kuoshui	97524, 97525	0	2	R	O
Dicaeidae	*Dicaeum concolor*	Shiwandashan	96457, 96458, 96459, 96460, 98124, 98127	0	6	R	F
Dicruridae	*Dicrurus hottentottus*	Jing Xi	93401, 93402, 96624, 97113, 97115	0	5	R	F
		Shiwandashan	96419	0	1	R	F
	D. leucophaeus	Jing Xi	93397, 93398, 93399	0	3	M	O
Emberizidae	*Emberiza elegans*	Dashahe	97431, 98929, 98950, 99314, 99316, 99349	0	6	M	O
		Kuan Kuoshui	97532, 97533, 97534, 97535,† 97536, 97537, 97538, 97607, 97608,† 99307	2	10	M	O
	E. godlewskii	Dashahe	97430	0	1	R	O
	E. pusilla	Dashahe	97422	0	1	M	O
		Kuan Kuoshui	97527,† 97528, 97529, 97530, 98918	1	5	M	O

		Shuipu	99401, 99859, 99860, 99861, 99862, 99965, 99981	0	7	M	O
	E. spodocephala	Dashahe	97429, 98939	0	2	M	O
		Kuan Kuoshui	97539, 97540, 99306	0	3	M	O
		Shuipu	99439, 99863, 99864, 99865, 99866, 110308, 1 not catalogued	0	7	M	O
	E. tristrami	Shuipu	99858	0	1	M	F
	Latoucheornis siemsseni	Shuipu	99452, 99851	0	2	M	O
	Melophus lathami	Shuipu	99849, 99850,110313	0	3	R	O
Estrilididae	*Lonchura striata*	Shuipu	99455, 99456, 99840, 99841, 99842, 99843, 99844, 99845, 99939, 99951, 99952, 110310, 1 not catalogued	0	13	R	O
Fringillidae	*Carduelis sinica*	Kuan Kuoshui	97541	0	1	M	O
	Carpodacus erythrinus	Shuipu	99415, 99416, 99846, 99847, 99848, 110318	0	6	M	O
Hirundinidae	*Hirundo daurica*	Shuipu	99748, 99749, 99750	0	3	M	O
Laniidae	*Lanius schach*	Shuipu	99688	0	1	R	O
Monarchidae	*Rhipidura albicollis*	Jing Xi	1 not catalogued, 93373, 93374, 93375, 96578, 98133	0	6	R	F
	Terpsiphone paradisi	Shiwandashan	96274, 96447, 96448, 96451, 96452, 96453, 96973, 97205	0	8	R	F
Motacillidae	*Anthus hodgsoni*	Dashahe	97427, 97428	0	2	M	O
		Jing Xi	96622	0	1	M	O
		Shuipu	99421, 99429, 99781, 99782, 99783, 99964, 99967, 99971, 110306, 110329	0	10	M	O
	Motacilla alba	Jing Xi	96620, 96621	0	2	M	O
		Kuan Kuoshui	97452	0	1	M	O
		Shuipu	110312, 110331	0	2	M	O
	M. cinerea	Dashahe	97479	0	1	M	O
	M. cinerea	Jing Xi	96619†	1	1	M	O
Muscicapidae	*Brachypteryx*	Dashahe	97485†	1	1	M	F

montana						
Copsychus saularis	Kuan Kuoshui	97522†	1	1	R	O
Culicicapa ceylonensis	Jing Xi	93336, 93338, 98131	0	3	R	F
	Kuan Kuoshui	97542, 97543, 98945, 98950	0	4	R	F
	Shuipu	99418, 99453, 99813, 99814, 99815, 99816, 99817, 99818, 99946, 99953, 99957, 110315, 110333, 3 not catalogued	0	16	R	F
Cyanoptila cyanomelana	Jing Xi	93333	0	1	M	F
	Shiwandashan	96520	0	1	M	F
Cyornis banyumas	Jing Xi	93327, 93328, 93329, 93330, 93331,† 93332, 97074	1	7	R	F
	Shiwandashan	96504, 96505, 96506, 96507, 96508, 98211	0	6	R	F
	Shuipu	99457, 99699, 99700, 99968	0	4	R	F
C. hainanus	Jing Xi	93343, 96574	0	2	R	F
	Kuan Kuoshui	97519, 97520, 97589	0	3	R	F
	Shiwandashan	96496, 96497, 96499, 96501, 96502, 96503	0	6	R	F
	Shuipu	110316, 110332	0	2	R	F
Enicurus leschenaulti	Kuan Kuoshui	97546, 97547, 97548	0	3	R	F
	Shuipu	99408, 99805, 99806	0	3	R	F
E. schistaceus	Jing Xi	93403, 97391	0	2	R	F
	Shiwandashan	96517, 96518, 97206	0	3	R	F
	Shuipu	99434	0	1	R	F
Eumyias thalassina	Dashahe	97499	0	1	M	O
	Kuan Kuoshui	97523	0	1	M	O
Ficedula hyperythra	Shiwandashan	96498	0	1	R	F
F. mugimaki	Shuipu	99704	0	1	M	O
F. parva	Jing Xi	93345	0	1	M	O

	Shiwandashan	96548	0	1	M	O
	Shuipu	99403, 99404	0	2	M	O
F. tricolor	Kuan Kuoshui	97471	0	1	M	O
	Shuipu	99406, 99705	0	2	M	O
F. zanthopygia	Shiwandashan	96549, 96550	0	2	M	O
Luscinia cyane	Jing Xi	93348	0	1	M	F
L. sibilans	Shuipu	99703	0	1	M	F
Muscicapa ferruginea	Jing Xi	93370, 93371, 97109, 98143	0	4	M	F
M. muttui	Dashahe	97496, 97497, 97498	0	3	M	F
	Jing Xi	93369, 96571, 97476	0	3	M	F
	Shiwandashan	96493, 96494	0	2	M	F
	Shuipu	99702	0	1	M	F
Myiomela leucura	Kuan Kuoshui	97549, 97550, 97551, 99341	0	4	R	F
	Shiwandashan	96519, 96521	0	2	R	F
Myophonus caeruleus	Jing Xi	96396	0	1	R	F
	Kuan Kuoshui	97521	0	1	R	F
	Shuipu	99831, 99832, 99936, 99970	0	4	R	F
Niltava davidi	Dashahe	97514	0	1	M	F
	Kuan Kuoshui	1 not catalogued, 97515, 97516, 97517, 99310, 99340	0	6	M	F
	Shuipu	99692,† 99693, 99694, 99695, 99695, 99696, 99697, 99698, 110335	1	9	M	F
N. grandis	Jing Xi	93339, 93340, 96577, 98219	0	4	M	F
N. macgrigoriae	Jing Xi	3 not catalogued, 93342, 96580, 96581,† 96583, 97079, 97080, 98138	1	10	R	F
	Shiwandashan	96509	0	1	R	F
Phoenicurus auroreus	Dashahe	99361	0	1	M	O

		Kuan Kuoshui	97544, 97545	0	2	M	O
	Rhyacornis fuliginosus	Dashahe	97489, 97491, 97492, 97493, 97494, 97495, 97624, 98930, 99312, 99352†	1	10	R	F
		Shiwandashan	96513, 96514	0	2	R	F
	Saxicola ferrea	Dashahe	97500	0	1	M	O
		Jing Xi	96588	0	1	M	O
		Shuipu	99411	0	1	M	O
	Zoothera citrina	Jing Xi	93407, 93408	0	2	M	F
		Shiwandashan	96387, 96388, 96391, 96392	0	4	M	F
		Shuipu	99407, 99835, 99836, 99837, 1 not catalogued	0	5	M	F
	Z. dauma	Shuipu	99833, 99834, 99937	0	3	M	F
Nectariniidae	*Aethopyga christinae*	Shiwandashan	96462, 96463, 96465, 96466, 98122, 98210	0	6	R	O
		Shuipu	99422, 1 not catalogued	0	2	R	O
	A. gouldiae	Kuan Kuoshui	98914	0	1	R	M
	Arachnothera magna	Shiwandashan	96547	0	1	R	O
Panuridae	*Paradoxornis alphonsianus*	Kuan Kuoshui	97450, 97470, 99303	0	3	R	F
	P. gularis	Jing Xi	96627	0	1	R	F
		Kuan Kuoshui	97451	0	1	R	F
	P. verreauxi	Kuan Kuoshui	97447, 97448, 97449, 97600, 97601, 98915, 99299	0	7	R	F
	P. webbianus	Dashahe	97482, 97483	0	2	R	O
		Shuipu	99440, 99766, 99767, 99768, 99769, 99962	0	6	R	O
Paridae	*Melanochlora sultanea*	Jing Xi	93412	0	1	R	F
	Parus major	Kuan Kuoshui	97463, 98911	0	2	R	O
		Shuipu	99412, 99757, 99758, 99959	0	4	R	O
	P. monticolus	Dashahe	97484, 99313	0	2	R	O
		Kuan Kuoshui	97464, 97465, 97466, 97467, 97468, 97588, 97595,	0	16	R	O

			97596, 97597, 98907, 99301, 99308, 99309, 99311, 99335, 99348				
	P. venustulus	Shuipu	99432, 99754, 99755, 99756, 99940, 99960	0	6	R	O
	Sylviparus modestus	Kuan Kuoshui	97469,† 97605	1	2	R	F
Passeridae	*Passer rutilans*	Dashahe	97425†	1	1	R	O
		Kuan Kuoshui	97531†	1	1	R	O
		Shuipu	99425, 99425, 99426, 99853, 99854, 99855, 99856, 99857, 99972, 110305, 1 not catalogued	0	11	R	O
Pycnonotidae	*Alophoixus pallidus*	Jing Xi	93420, 96616, 96617, 98128	0	4	R	F
		Shiwandashan	96432, 96434, 96436, 96975, 98209	0	5	R	F
	Hemixos castanonotus	Shiwandashan	96437, 96441, 96442, 96444, 97201	0	5	R	F
		Shuipu	99438, 99738, 99739, 99740, 99741, 99742, 99743, 99949, 99961, 110311, 2 not catalogued	0	12	R	F
	Hypsipetes leucocephalus	Shiwandashan	96426	0	1	R	F
		Shuipu	99745	0	1	R	F
	H. mcclellandii	Dashahe	97416, 99360	0	2	R	F
		Jing Xi	93417, 93418, 96613	0	3	R	F
		Kuan Kuoshui	97402, 97403, 97404	0	3	R	F
		Shiwandashan	96454, 96455, 96456	0	3	R	F
		Shuipu	99744, 110309, 110328, 1 not catalogued	0	4	R	F
	Pycnonotus jocosus	Jing Xi	96618	0	1	R	O
		Shiwandashan	97202, 96429, 96430	0	3	R	O
	P. sinensis	Shiwandashan	96427	0	1	R	O
		Shuipu	99728, 99729, 99730	0	3	R	O
	P. xanthorrhous	Dashahe	97417, 97418	0	2	R	O
		Kuan Kuoshui	97405	0	1	R	O
		Shuipu	99435, 99731, 99732, 99734, 99735, 99736, 99737, 99954, 110307, 2 not catalogued	0	11	R	O

	Spizixos semitorques	Dashahe	97419, 97420, 98920, 99350, 99362	0	5	R	O
		Shuipu	99417, 99427, 99428, 99718, 99719, 99720, 99721, 99722, 99723, 99724, 99727, 110314, 110325, 110334, 2 not catalogued	0	16	R	O
Sturnidae	*Acridotheres cristatellus*	Dashahe	97423	0	1	R	O
Sylviidae	*Cettia fortipes*	Kuan Kuoshui	1 not catalogued, 97462,† 99305	1	3	R	F
		Shuipu	99803	0	1	R	F
	Orthotomus cucullatus	Jing Xi	93356	0	1	R	O
	O. sutorius	Shiwandashan	96525	0	1	R	O
	Phylloscopus sp.	Dashahe	97366, 97367, 97368	0	3	M	F
		Shuipu	4 not catalogued	1	4	M	F
	P. coronatus	Shiwandashan	96540	0	1	M	F
		Jing Xi	96560	0	1	M	F
	P. fuscatus	Shiwandashan	96542	0	1	M	F
	P. inornatus	Kuan Kuoshui	97365	0	1	M	F
		Shuipu	99822	0	1	M	F
	P. proregulus	Shuipu	99819, 99820, 99821	0	3	M	F
	P. reguloides	Dashahe	98928,† 97364, 97615, 97616, 97617, 98932	1	6	M	F
		Jing Xi	96559,† 96555, 96556, 96557, 96558	1	5	M	F
		Kuan Kuoshui	97359, 97361, 97362	0	3	M	F
		Shuipu	99823, 99825, 99824, 99826	0	4	M	F
	P. ricketti	Jing Xi	93362, 93363, 93364, 93365, 96954, 98137	0	6	M	F
		Shiwandashan	96527, 96528, 98126	0	3	M	F
	P. schwarzi	Kuan Kuoshui	97369†	1	1	M	F
	P. tenellipes	Jing Xi	93361, 96553	0	2	M	F
		Shiwandashan	96541, 96543, 96544, 96546	0	4	M	F
	Prinia atrogularis	Shiwandashan	96523, 96524	0	2	R	O

		Shuipu	99807	0	1	R	O
	P. inornata	Shuipu	99809	0	1	R	O
	Seicercus sp.	Kuan Kuoshui	97611, 97612, 98475	0	3	R	F
		Dashahe	97613, 97614	0	2	R	F
	S. burkii	Dashahe	98912, 98920, 98921, 98923, 98924, 98925, 98926, 98927, 99355	0	9	R	F
		Jing Xi	93355, 96566, 96568, 97073, 97377, 4 not catalogued	0	9	R	F
		Kuan Kuoshui	97370, 97371, 97372, 97373, 98906, 98909, 99337, 99344	0	8	R	F
		Shiwandashan	96531	0	1	R	F
	S. affinis	Shuipu	99827, 99828, 99829, 99830, 1 not catalogued	0	5	M	F
	S. castaniceps	Kuan Kuoshui	97459, 97460	0	2	R	F
	S. poliogenys	Jing Xi	93352, 93353	0	2	R	F
	Tesia cyaniventer	Jing Xi	93357, 93358, 93359, 96561, 97110	0	5	R	F
	Urosphena squameiceps	Jing Xi	93366, 93367, 96563	0	3	M	F
Timaliidae	*Alcippe chrysotis*	Dashahe	97434	0	1	R	F
		Kuan Kuoshui	97435, 97436, 97437, 97440, 97604, 98905, 99298	0	7	R	F
	A. dubia	Dashahe	97477	0	1	R	F
		Shuipu	99402	0	1	R	F
	A. morrisonia	Dashahe	98935, 99318, 99356, 99357	0	4	R	F
		Jing Xi	93377, 93378, 93379, 93380, 96589, 5 not catalogued	0	10	R	F
		Kuan Kuoshui	97441, 97442, 97443, 97444, 97445, 97446, 97583, 97585, 97586,† 97593, 97594, 98916, 98917, 99297, 99300,† 99304, 99338, 99339, 2 not catalogued	2	20	R	F
		Shiwandashan	96471, 98125	0	2	R	F
		Shuipu	99442, 99443, 99444, 99445, 99446, 99449, 99706, 99707, 99708, 99709, 99710, 99711, 99712, 99713, 99714, 99715, 99717, 16 not catalogued	0	33	R	F

Babax lanceolotus	Dashahe	97501, 97502, 97623, 98941	0	4	R	O
Garrulax canorus	Shiwandashan	1 not catalogued	0	1	R	O
G. chinensis	Jing Xi	96400	0	1	R	F
	Shiwandashan	96401	0	1	R	F
G. cineraceus	Kuan Kuoshui	97566, 97567,† 97590, 98948	1	4	R	F
	Shuipu	99420, 99751, 99752, 99753	0	4	R	F
G. milnei	Jing Xi	93390	0	1	R	F
	Kuan Kuoshui	97555, 97556, 97557, 97558, 97559, 97560, 97561, 97562, 97563, 97609	0	10	R	F
G. ocellatus	Dashahe	97504	0	1	R	F
G. pectoralis	Kuan Kuoshui	97554, 99343	0	2	R	F
G. poecilorhynchus	Kuan Kuoshui	97552, 97553, 97610	0	3	R	F
G. sannio	Kuan Kuoshui	97568, 97569, 97570	0	3	R	O
	Shuipu	99414	0	1	R	O
Minla cyanouroptera	Dashahe	97505	0	1	R	F
	Kuan Kuoshui	97572, 97573, 97587, 97599, 98951, 99346	0	6	R	F
M. ignotincta	Dashahe	97506, 97507	0	2	R	F
	Kuan Kuoshui	97571	0	1	R	F
Napothera brevicaudata	Jing Xi	93349, 93350, 93351, 96607, 96608, 97392	0	6	R	F
	Shiwandashan	96484	0	1	R	F
Pellorneum albiventre	Jing Xi	93321, 93323, 96610	0	3	R	F
	Shiwandashan	96974	0	1	R	F
P. tickelli	Jing Xi	93324	0	1	R	F
Pomatorhinus ruficollis	Dashahe	97511, 97512, 97513	0	3	R	F
	Jing Xi	93413, 93414, 93415, 96592, 97390, 98144	0	6	R	F
	Kuan Kuoshui	97370, 97576, 97578, 97579, 97580, 97581, 97582, 99333, 1 not catalogued	0	9	R	F

		Shiwandashan	96475, 96476, 96476	0	3	R	F
		Shuipu	99451, 99770,† 99771, 99772, 99773, 99774, 99775, 99777, 99778, 99779, 99780, 2 not catalogued	1	13	R	F
	Pteruthius flaviscapis	Jing Xi	93410	0	1	R	F
	Stachyris nigriceps	Jing Xi	93382, 96593 96594, 96597, 96598, 96599, 96983, 97070, 97108,† 98129, 98215, 98216, 98217, 6 not catalogued	1	19	R	F
	S. ruficeps	Dashahe	99358	0	1	R	F
		Jing Xi	93385	0	1	R	F
		Kuan Kuoshui	97455, 97456, 98919, 99318, 99342, 99356, 99356, 1 not catalogued	0	8	R	F
		Shiwandashan	6 not catalogued	0	6	R	F
		Shuipu	99419, 99430, 99431, 99790, 99791, 99792, 99793, 99794, 99795, 99796, 99797, 99798, 99947, 110327, 3 not catalogued	0	17	R	F
	S. striolata	Jing Xi	93383, 93384, 96600, 96603, 96605, 2 not catalogued	0	7	R	F
		Shiwandashan	96481	0	1	R	F
	Yuhina castaniceps	Shiwandashan	96535, 96536, 96537, 96538, 96539, 98213	0	6	R	F
		Shuipu	99410, 99799, 99800, 110330	0	4	R	F
	Y. diademata	Kuan Kuoshui	97574	0	1	R	O
	Y. nigrimenta	Dashahe	97508, 99360	0	2	R	O
		Kuan Kuoshui	97575, 99347	0	2	R	O
		Shuipu	99969	0	1	R	O
	Y. zantholeuca	Jing Xi	93387, 93388, 93389, 97069, 97117, 98139, 98142, 1 not catalogued	0	8	R	F
		Shiwandashan	96532, 96533, 96534	0	3	R	F
		Shuipu	99801, 99802	0	2	R	F
Turdidae	*Turdus dissimilis*	Shuipu	99413, 99838	0	2	R	O
Zosteropidae	*Zosterops japonicus*	Kuan Kuoshui	98952†	1	1	M	O

			Shiwandashan	96551	0	1	M	O
			Shuipu	99448, 99784, 99786, 99787, 99788, 99789, 99938, 110317, 110336, 2 not catalogued	0	11	M	O
Piciformes	Capitonidae	*Megalaima franklinii*	Shiwandashan	96405	0	1	R	F
	Picidae	*Dendrocopos canicapillus*	Kuan Kuoshui	97410	0	1	R	F
			Shuipu	99682	0	1	R	F
		Picumnus innominatus	Dashahe	97412	0	1	R	F
			Kuan Kuoshui	97411, 97591, 98947	0	3	R	F
			Shuipu	99405, 99678, 99679, 99680, 99966	0	5	R	F
		Picus canus	Kuan Kuoshui	97408, 97409	0	2	R	F
		Sasia ochracea	Jing Xi	93394, 93395, 93396, 96630, 97111	0	5	R	F
			Shiwandashan	96413, 96415, 96416, 98208	0	4	R	F
			Shuipu	99437, 99681, 99942, 99942, 99958	0	5	R	F
Podicipediformes	Podicipedidae	*Tachybaptus ruficollis*	Kuan Kuoshui	97401	0	1	M	O
Trogoniformes	Trogonidae	*Harpactes erythrocephalus*	Jing Xi	93311	0	1	R	F

*The Kansas University Natural History Museum (KUNHM) provided the permanent catalog number of the specimen to which the individual specimen can be referred. M, migratory; O, open country; R, resident; F, forested; not catalogued, specimens not catalogued at KUNHM.

†Individual birds infected with avian influenza virus.
